# Clinical/Sonographic Assessment and Management of Calcific Tendinopathy of the Shoulder: A Narrative Review

**DOI:** 10.3390/diagnostics12123097

**Published:** 2022-12-08

**Authors:** Vincenzo Ricci, Kamal Mezian, Ke-Vin Chang, Levent Özçakar

**Affiliations:** 1 Physical and Rehabilitation Medicine Unit, Luigi Sacco University Hospital, ASST Fatebenefratelli-Sacco, 20157 Milan, Italy; 2 Department of Rehabilitation Medicine, First Faculty of Medicine and General University Hospital, Charles University, 12800 Prague, Czech Republic; 3 Department of Physical Medicine and Rehabilitation, National Taiwan University Hospital, Bei-Hu Branch, Taipei 10845, Taiwan; 4 Department of Physical and Rehabilitation Medicine, Hacettepe University Medical School, 06100 Ankara, Turkey

**Keywords:** rotator cuff, ultrasonography, tendon, calcification, migration

## Abstract

Shoulder disorders are very common in clinical practice. Among several other pathologies, calcific tendinopathy of the rotator cuff tendons is frequently observed during the ultrasound examination of patients with painful shoulder. The deposition of hydroxyapatite calcium crystals should not be considered as a static process but rather a dynamic pathological process with different/possible patterns of migration. In this paper, we have illustrated how and where these calcium depositions can migrate from the rotator cuff tendons to the peri-articular soft tissues. We have also tried to discuss the issue from the clinical side, i.e., how these particular conditions might impact the specific diagnosis, appropriate rehabilitation plan or interventional approach for optimal functional recovery.

## 1. Introduction

Shoulder disorders are commonplace in daily practice and calcific tendinopathy of the rotator cuff tendons can be the main finding of an ultrasound (US) examination [1]. In calcific tendinopathy, deposition of hydroxyapatite is a dynamic pathological occurrence with different patterns of clinical presentation. Identifying these patterns through US can provide additional insight into this condition as well as optimizing its management [2,3].

The pathogenesis appears to stem from a low amount of oxygen within the tendons, leading to fibrocartilaginous metaplasia, i.e., switch of tenocytes to chondrocytes. The latter cellular line produces a cartilaginous matrix that progressively calcifies [4,5]. Indeed, the rotator cuff tendons are poorly vascularized and receive nutrients mainly from the overlying synovial bursa [4,5]. The *primum movens* of the pathology could be a “metabolic disorder” characterized by reduced passage of nutrients/oxygen from the vascular network located within the peri-bursal fat tissue to the underlying tendons.

**Figure 1:**
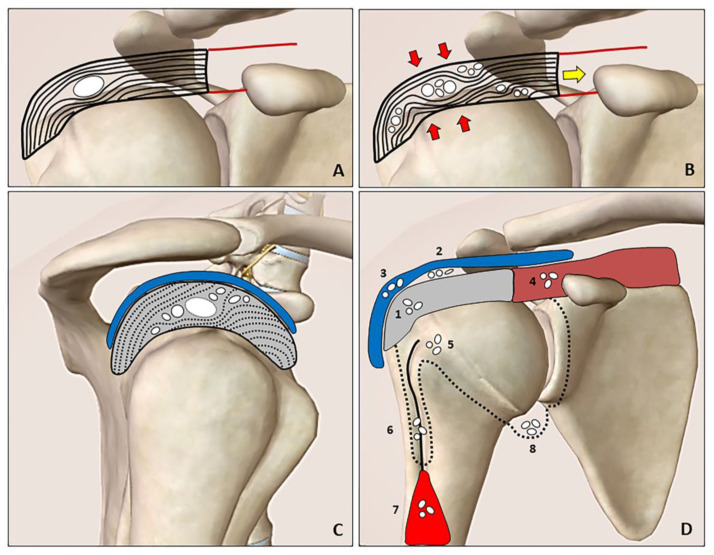
The resting phase is characterized by a hard calcification that shifts the tendon fibers (**A**). During the resorptive phase (**B**), the compression (*red arrows*) and tension (*yellow arrow*) forces promote slipping of the softly hydrated calcification through the layers/laminae of the tendon (rupture and dispersion of the calcific deposition). The intratendinous bursal-side (cranial direction) and articular side (caudal direction) migration patterns can be clearly identified during the US imaging by “following” the hyperechoic foci of the calcification (*white dots*) especially in the short axis view (**C**). Hydroxyapatite calcium crystals (*white dots*) can develop “migratory patterns”, i.e., moving from the rotator cuff tendons (*grey*) to several anatomical sites. The spectrum includes the following patterns: intratendinous *(1)*, sub-bursal (*2*), intrabursal (*3*), intramuscular (*4*), intraosseous (*5*), inside the synovial sheat of the long head of the biceps tendon (i.e., the bicipital recess) (*6*), between the interfascial planes of the arm (*7*) and perhaps inside the glenohumeral joint cavity (*8*) (**D**). Blue: subacromial bursa, brown: supraspinatus muscle, red: biceps brachii muscle, black line: long head of the biceps tendon, black dotted line: synovium.

The natural history of calcific tendinopathy is characterized by precalcific, calcific and postcalcific stages (Uhthoff cycle). Moreover, the calcific stage is further divided into formative, resting (Figure 1A) and resorptive phases (i.e., maturation process) [4,5]. Calcifications display distinct patterns of migration during the maturation process during which hydroxyapatite calcium crystals may migrate from tendons to neighboring tissues under mechanical (tension/compression) forces. The fragments can creep through the intrasubstance gaps of the tendon where the layers/laminae are not tightly attached to each other (delaminating zones) (Figure 1B).

Calcium deposition may be extruded from the tendon cranially towards the sub-bursal space and subacromial bursa (i.e., intra-bursal penetration with acute microcrystalline bursitis), or caudally towards bone/synovium of the joint (Figure 1C). The most commonly described patterns are intratendinous, sub-bursal, intrabursal, intramuscular, intraosseous and intraarticular migrations (Figure 1D) [2,3]. Interfascial migration of softly hydrated calcification slipping from the originating tendon over the superficial fascia of biceps brachii muscle was also described [6]. An intra-articular migration has never been demonstrated by imaging modalities; however, several authors have reported the frequent onset of shoulder stiffness after arthroscopy for calcific tendinopathy—postulating the irritation of the glenohumeral capsule by residual calcium debris as the possible mechanism [7]. Likewise, we speculate that chronic micro-leakage of calcium, moving from the rotator cuff tendons inside the synovial recesses of the joint (e.g., axillary recess due to gravity) (Figure 1D), could increase the risk of stiffness through inflammatory involvement of the capsulo-synovial complex (i.e., post-calcific frozen shoulder).

In daily practice, understanding the correlation between the clinical features of the patient and the ultrasonographic pattern of calcific tendinopathy is paramount to plan for an appropriate/substantial rehabilitation program (conservative and interventional alike). In this sense, we briefly describe the most common clinical-ultrasonographic scenarios of calcific tendinopathy of the shoulder in different phases/stages.

## 2. Materials and Methods

In order to develop a practical, ready-to-use guide for the management of patients with calcific tendinopathy of the shoulder in daily practice, the authors have planned a 3-step workflow as described below:

1st Phase: an extensive review of the scientific literature on shoulder calcific tendinopathy has been performed, mainly focusing on multiple clinical scenarios and sonographic patterns related to different disease stages. PubMed and Web of Science were searched using the following keywords: “ultrasound”, “sonography”, “shoulder”, “calcific tendinopathy” and “phases”.

2nd Phase: the clinical scenarios and sonographic patterns of the different stages of shoulder calcific tendinopathy have been matched using systematic description of stages (i.e., resting, resorptive, post-calcific) stages.

3rd Phase: an additional review of the pertinent literature has been performed to describe the most suitable therapeutic approach for each and every phase of shoulder calcific tendinopathy. Of note, conservative treatments as well as US-guided interventional (but not surgical) procedures have been included in the present manuscript. In this regard, “conservative treatment”, “ultrasound-guided”, “procedure”, “injection” and “intervention” have been used as the keywords to explore studies describing a specific intervention/treatment for a specific phase of the disease.

## 3. Results and Discussion

Considering the particular nature of the present review, the authors have combined the results and discussion sections to provide a unique phase-by-phase description of rotator cuff calcific tendinopathy. For each and every stage of the aforementioned disease, typical/main clinical hallmarks, sonographic patterns and potential therapeutic approaches have been described to optimize the practical management of patients in daily practice (Table 1).

### 3.1. Resting Phase

**Figure 2:**
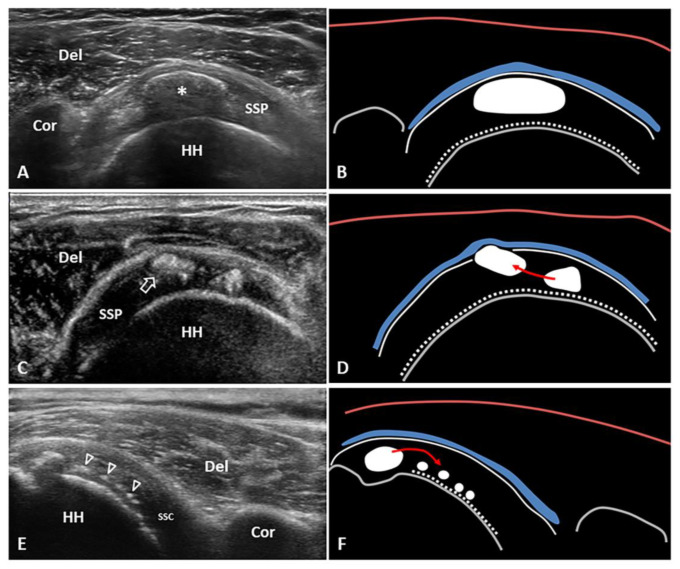
Short-axis view of the supraspinatus (*SSP*) tendon with elliptical calcification (*asterisk*) in resting phase—i.e., type 1 Gartner and Heyer (**A**,**B**). Short-axis view of the SSP tendon with intratendinous bursal side migration pattern of a softly hydrated fragment (*white void arrow*) of calcific deposits with sub-bursal space involvement (**C**,**D**). Long-axis view of the subscapularis (*SSC*) tendon with intratendinous articular side migration pattern of multiple hydroxyapatite calcium crystals (*white void arrowheads*) towards the surface of the humeral head (**E**,**F**). The latter might be a “migratory pattern” possibly predisposing to ‘post-calcific’ frozen shoulder. Del: deltoid, Cor: coracoid, HH: humeral head, blue lines: subacromial bursa, white dotted lines: cartilage, white lines: outer surface of the rotator cuff tendons, red lines: superficial fascia of the deltoid muscle, grey lines: bony surface, red arrow: possible direction of migration.

Hard calcification located between the tendon fibers (i.e., displacing them) is associated with focal thickening of the rotator cuff (Figure 2A,B). The possible mechanical impingement to the surrounding bones, ligaments and muscles during motions is referred as the *mechanical phase* of calcific tendinopathy.

The specific movements causing pain depend on the anatomical location of the hard (calcific) deposit. In this sense, a calcification located inside the supraspinatus tendon may be impinged under the coracoacromial arch during abduction, unlike a calcification inside the subscapularis tendon which may conflict anteriorly with the coracoid bone (or with the muscles attached to the coracoid process) during rotations of the glenohumeral joint. Of note, by coupling the clinical tests and sonographic findings, non-specific definitions such as subacromial impingement or anterior impingement of the shoulder can be promptly replaced with the exact description of the pathological condition, also guaranteeing reproducibility of the examination. For instance, a generic diagnosis of subacromial impingement can be replaced by calcific tendinopathy of the pre-insertional zone of the supraspinatus tendon in the resting phase, impinging the coracoacromial ligament during dynamic scanning—reproducing the painful complaint of the patient as well.

The resting phase of calcific tendinopathy is usually characterized by a movement-induced pain of the shoulder with possible feeling of ‘clicks’ when the intra-tendinous calcification snaps under the surrounding soft tissues (Table 1) [8]. During this phase, management of the patient is usually focused on decoaptation exercises to reduce the mechanical conflict—e.g., Codman’s (pendulum) exercises in case of subacromial impingement—and on rebalancing of the force vectors involved in correct shoulder movements [9]. Typically, specific strengthening exercises for the humeral head depressor muscles—latissimus dorsi and the inferior fibers of pectoralis major (long depressors), and the inferior fibers of the subscapularis muscle (short depressor)—are part of the rehabilitation program in case of impingement under the acromion. They prevent excessive cranial translation of the humeral head by the deltoid muscle [10]. Closed kinetic chain exercises are usually preferred for the first phase of the rehabilitation training with a progressive shift to open kinetic chain movements. Moreover, isometric exercises for the tonic component of the muscle fibers should be coupled with isotonic exercises in order to also train the phasic component [11].

A frequent complication that occurs during the resting phase of calcific tendinopathy, especially if not properly treated, is the development of bursopathy due to excessive frictions between the thickened portion of the rotator cuff (in which the hard calcification is located) and the overlying synovial bursa. Indeed, the sonographic pattern of bursal pathology can be extremely variable, ranging from typical exudative bursitis with anechoic effusion to “dry” bursitis (adhesive bursopathy). It is more challenging to recognize/diagnose the latter, because it often presents with only mild, nodular thickening of the synovial walls and can be overlooked especially by beginners [12]. In case of bursitis, US-guided injection of the involved synovial bursa is paramount to correctly pursuing the rehabilitation process with satisfactory pain control [12,13].

The authors suggest using a high-volume (8 to 10 mL) injection in patients with a clinical/sonographic pattern of adhesive bursopathy in order to effectively break up the intra-bursal adhesions—optimizing the clinical efficacy of the US-guided procedure. In the pertinent literature, high-volume compared to low-volume US-guided injections of the SASD bursa have been found superior for achieving early pain recovery and increasing the chances of long-term symptom remission in patients with subacromial impingement, rotator cuff tendinopathy, and shoulder overuse [14]. Interestingly, bursal adhesions not only lead to increase of subacromial pressure; they also progressively induce fibrotic involution of the synovial bursa which acts as a resilient force during shoulder abduction via drawing the humeral head towards the acromion—i.e., promoting the impingement mechanism [15]. Of note, in case of impingement-induced bursitis, the pain acquires inflammatory features and may also ensue at rest (i.e., not only during motions as in the mechanical type).

Lastly, the ultrasonographic pattern of hard calcification with complete acoustic shadowing (as in the resting phase of calcific tendinopathy) (Figure 2A,B) corresponds to ‘type 1’ of the radiological classification by Gartner and Heyer (i.e., well circumscribed and dense calcification). At this stage, the calcific deposit responds poorly to extracorporeal shockwave therapy [16]. Likewise, some authors have also proposed fine-needle repeated perforations (needling) of the hard, arc-shaped calcific plaque to promote/accelerate the transition from the resting phase to the resorptive phase of the Uhthoff cycle. The mechanism is believed to be via inducing local hyperemia and recruitment of phagocytes [17].

### 3.2. Resorptive Phase

During the resorptive phase, intra-tendinous calcific deposition usually breaks down and the sonographic pattern can be highly variable—e.g., fragmented, nodular, cyst-like [18]. Moreover, color/power Doppler signals can be identified surrounding the hydroxyapatite crystals due to the local proliferation of capillaries and thin-walled vascular channels [18,19]. Of note, the aforementioned intratendinous neovessels allow macrophages and multinucleated giant osteoclast-like cells to reach and degrade the calcific deposits [19].

**Figure 3:**
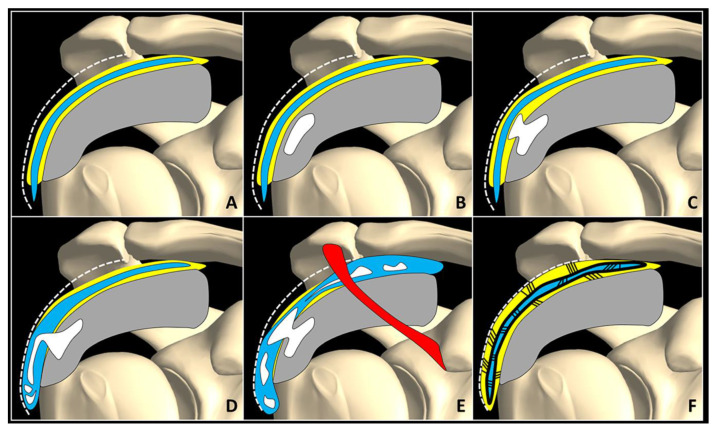
Normally, fat tissue (*yellow*) is located in between the SASD bursa (*blue*), rotator cuff tendons (*grey*), and the subdeltoid fascia (*white dotted line*) (**A**). Intra-tendinous calcification (*white*) (**B**) may progressively migrate within the peribursal space (**C**), and perforating the synovial lining, may slip inside the bursal cavity involving its lateral recess (**D**) or the entire chamber (**E**). A potential complication of acute microcrystalline bursitis is adhesive bursopathy (**F**) characterized by thickening of the synovial walls (*thick black line*) and intra/peri-bursal adhesions (*thin black lines*). Adhesions reduce the bursal gliding, “gluing” it to the rotator cuff tendons and subdeltoid fascia. Red: coracoacromial ligament.

If the fragments of hydroxyapatite crystals cranially migrate towards the peribursal fat (Figure 2C,D) and/or inside the subacromial-subdeltoid bursa (Supplementary Figure S1), acute microcrystalline bursitis may develop with a clinical scenario of shoulder hyperalgesia [20]. Intrabursal migration of calcific debris may predominantly involve the lateral recess of the synovial bursa; or, it can present a wide diffusion inside the bursal cavity. The first pattern is usually characterized by a teardrop-shaped bursal effusion located deep in the fibers of the deltoid muscle with pain on the lateral side of the shoulder. The second scenario is commonly that of as an hourglass-shaped bursal effusion with pain spreading throughout the shoulder (Figure 3). Histological studies have shown massive cell infiltration—mainly composed of polymorphonuclear cells and mononuclear phagocytic cells—which occurs within the whole bursal cavity and the synovial lining in response to hydroxyapatite crystal stimulation [21]. The patient typically complains of a pseudoparalytic shoulder with the upper limb adducted to the trunk in order to protect the shoulder and reduce pain [22]. Night rest is severely compromised and the patient is unable to lie on the affected side. In some patients, the sonographic visualization of intrabursal migration of calcific fragments can be challenging; dynamic scanning with gentle active/passive movements of the shoulder can be necessary to promptly identify “bright spots” floating within the bursal cavity [23].

During active resorption of the calcification, the patient is usually not able to perform therapeutic exercises due to excessive pain. In this sense, a US-guided intervention/procedure is often necessary to control the pain and allow the subsequent phases of the rehabilitation program (Table 1). US-guided corticosteroid injection of the subacromial bursa and US-guided barbotage (needling and lavage) of the calcific deposition are the two main therapeutic options in this phase—with no significant differences in clinical and radiological outcomes in the long-term (5 years) follow up [24]. In light of the authors’ experience, a 2-step procedure, i.e., US-guided lavage of the intra-tendinous calcific deposit (1st step) followed by corticosteroid injection of the subacromial bursa (2nd step), would be considered as the best approach for this phase of calcific tendinopathy. Indeed, while the former aims to reduce the intra-tendinous pressure and promote/accelerate the clearance of hydroxyapatite crystals from the rotator cuff tendons, the latter is usually performed to reduce the risk of post-procedural bursitis which is likely to ensue due to some fragments of the calcification (commonly) migrating within the subacromial bursa. Notably, the ultrasonographic pattern of well-demarcated homogeneous hyperechoic calcification with weak posterior acoustic shadowing (Figure 2C) indicates deposition with “soft texture” (type 2 Gartner and Heyer) that is suitable for US-guided percutaneous irrigation [25].

In the literature, two main techniques have been described for performing US-guided percutaneous irrigation. A single-needle technique in which lavage of the soft deposit is performed by pushing the syringe plunger to hydrate the calcification, and then aspirating the calcium debris with the help of the saline solution via the same syringe, as the plunger is released [26]. As regards the two-needle technique, the first needle is inserted within the lowest portion of the calcific deposit with the opening hole directed towards the probe. Then, the second needle is inserted inside the calcification, parallel and superficial to the first one but with its opening hole opposite to the first needle [26]. Respecting these positions, saline solution can be introduced inside the core of the calcific deposit through the first needle and removed using the second one—i.e., a washing circuit [26]. Of note, no significant differences have been identified between the single and double-needle techniques in terms of short and long-term clinical outcomes, post-procedural bursitis, ease of calcium dissolution, and overall procedure duration [27]. The authors suggest to accurately choose the thickness of the needle (18–20 G) in order to avoid unintentional filling of the lumen by the calcium debris during the lavage.

Extracorporeal shockwave therapy can also be considered as a potential treatment option in the resorptive phase of calcific tendinopathy (type 3, according to the radiological classification by Gartner and Heyer)—by inducing local hyperemia/neovascularization and leukocyte chemotaxis necessary to promote the phagocytosis of fragmented calcific deposits [16].

When the hyperalgesic phase is resolved, a tailored rehabilitation program with a combination of passive and active movements of the glenohumeral joint in different spatial planes must be performed to avoid the development of adhesive phenomena between the two synovial layers of the bursa and the surrounding peribursal fat tissue. The post-calcific adhesive bursopathy is historically known as *adhesive periarthritis* (Figure 3) [12,28,29,30]. Anatomical and radiological studies have clearly demonstrated partial or complete obliteration of the peribursal fat plane—due to inflammatory processes—in patients with calcific tendinopathy of the rotator cuff [28].

The acromiohumeral interval is a peculiar anatomical region of the shoulder where several soft tissues are located in between the deep surface of the deltoid muscle and the superficial portion of the rotator cuff, i.e., fat tissue, lax connective tissue, SASD synovial bursa and subdeltoid fascia. The latter is highly innervated/vascularized and presents a histological continuum with the coracoacromial ligament proximally and the periosteum of the humerus distally. Detailed knowledge of the aforementioned anatomical details is paramount to accurately interpret the sonographic findings of the resorptive phase of calcific tendinopathy.

According to the authors’ experience, some patients may complain of pain flare-up during the rehabilitation period, often related to the presence of residual calcific fragments tucked within the fat and loose connective tissue between the subdeltoid bursa and the rotator cuff tendons (possibly having migrated after the US-guided lavage). In this sense, if clinically indicated, US-guided needling of the residual fragments can be promptly performed to guarantee pain-free rehabilitation. Interestingly, the above quoted atypical location of small calcific deposits within the subbursal space have been historically described as the most challenging—for diagnosis and treatment alike [30]. Lastly, after the resorptive phase and in the portion of the tendon previously occupied by the calcific deposition, surgical and histological samples have demonstrated the presence of focal areas of granulation tissue with newly formed capillaries that are progressively replaced by foci of mature fibroblasts and neo-collagen fibrils [5]. In this regard, no tendon sequelae—e.g., partial/complete tear or loss of substance—can be sonographically observed after reabsorption of the calcific deposit [21].

### 3.3. Post-Calcific Stage

Even several months after resolution of the acute phase of calcific tendinopathy (e.g., resorptive phase) the patient may develop shoulder stiffness with or without pain [7,31]. The most probable cause of this clinical condition is adhesive capsulitis with thickening and fibrosis of the capsular connective tissue in the glenohumeral joint.

The pathophysiological link between calcific tendinopathy of the rotator cuff and adhesive capsulitis is not well understood; but chronic micro-leakage of calcium moving from the tendon fibers into the synovial recesses of the joint (Figure 2E,F) could be involved (i.e., chronic chemical synovitis) [32]. Interestingly, some authors have demonstrated that routine arthroscopic glenohumeral exploration performed before the calcification removal is associated with higher risk of post-operative adhesive capsulitis, probably related to the intra-articular diffusion of calcium debris coming from the rotator cuff tendons [33]. As such, the articular penetration of calcium—as a starter of synovial/capsular inflammation—can be considered to be a very likely link between the two shoulder disorders. The prolonged pain-induced hypomobility of the shoulder also seems to play important role in this aspect [7,31].

The main sonographic findings of adhesive capsulitis described in the literature are thickening of the axillary pouch (i.e., inferior capsular recess of the glenohumeral joint) and coracohumeral ligament in B-mode, and hypervascularization of the rotator cuff interval soft tissues in color/power Doppler mode [32,34]. Herein, the latter sonographic sign has been arthroscopically confirmed to be related to hyperemic synovial tissue surrounding the proximal segment of the long head of the biceps tendon (LHBT) within the rotator cuff interval [34]. Progressively, synovitis can evolve to synovial hypertrophy by replacement of the fat tissue located inside the rotator cuff interval with fibrous tissue. Hereby, US examination can reveal an irregular hypoechoic coat surrounding the proximal portion of the LHBT in more advanced stages of the disease [35].

Notably, capsular contracture tends to shift the articular effusion towards the bicipital and subcoracoid recesses of the shoulder, where the synovial tissue lacks the capsular coat and is more stretchable [13,36]. Moreover, dynamic and comparative US assessment—pathological vs. normal shoulder—can be performed to demonstrate the rotational blockade of the humeral head as well as the disappearance of physiological retroflection of the posterior glenohumeral recess under the infraspinatus muscle during external rotation [37].

During the post-calcific stiffness phase of the shoulder, the main purpose of rehabilitation is rapid recovery of the active and passive range of motions. The pertinent literature shows the efficacy of combined treatment with US-guided glenohumeral injection and rehabilitation training to accelerate the improvement in pain and function in case of adhesive capsulitis (Table 1) [38]. Likewise, some authors have also suggested using high-volume injections to mechanically expand the joint space (i.e., hydrodilatation or intra-articular hydraulic distension) to stretch the capsule and “break” the adhesions, i.e., a mechanical effect in addition to the pharmacological effect of the corticosteroid over the chronic glenohumeral synovitis [39]. Of note, concerning US-guided hydrodilatation, the anterior approach through the rotator cuff interval seems to be more effective (than the posterior approach targeting the glenohumeral recess) in reducing pain during shoulder movements [40]. Accordingly, for dilating the anterior capsule of the glenohumeral joint, the needle’s tip can be advanced within the histological interface between the LHBT and the stabilizing pulley (i.e., coracohumeral and superior glenohumeral ligaments) [41] or in the gap between the superior edge of subscapularis tendon and the proximal segment of the LHBT [42].

After the US-guided injection/hydrodilatation, immediate rehabilitation including passive mobilization of the glenohumeral joint in different spatial planes (i.e., angular and translational mobilizations) and the end-of-range capsular stretching of the shoulder is mandatory to progressively improve the active/passive range of motions [43]. As regards active exercises, muscle energy techniques with isometric contraction against an operator’s resistance (in a controlled direction/position) seems to be more effective to improve function and disability—when compared to other types of therapeutic exercises in shoulder adhesive capsulitis [44].

Of note, the capsular tissue is richly innervated; therefore, the authors suggest to accurately plan for progressive stretching, to minimize the onset of local/radiating pain along the anterolateral surface of the arm—i.e., C5 and C6 dermatomes. The latter component of pain is mainly related to the innervation of the rotator cuff interval by suprascapular and subscapularis nerves that are derived from the anterior branches of C5–C6 nerve roots [45,46]. For sure, in case of advanced stiffness of the shoulder, US-guided suprascapular nerve block can also be performed—to facilitate pain-free mobilization of the glenohumeral joint during the functional recovery [47].

## 4. Conclusions

Calcific tendinopathy of the rotator cuff is a challenging pathology of the shoulder which manifests several sonographic patterns and multiple clinical scenarios. Interestingly, depending on the specific phase of the calcific deposition, it can mime several other shoulder disorders. To the best of our knowledge, a practical guide describing “how” to recognize and manage different phases of the disease is lacking in the pertinent literature. In this sense, coupling the clinical and sonographic features in a unique comprehensive examination, this manuscript is likely to serve as a novel approach to this “pleomorphic” pathology (Table 1). In daily clinical practice, it is crucial to understand how a hard calcification (i.e., resting phase) of the supraspinatus tendon—that is impinged by the coraco-humeral arch causing mechanical pain during motions—requires different management from a soft calcification (i.e., resorptive phase) that has migrated into the subbursal space causing bursitis and nocturnal inflammatory pain (Figure 2). Last but not least, US follow-up performed by musculoskeletal physicians after specific interventions (e.g., injection, barbotage, therapeutic exercises) also allows simultaneous interpretation of ongoing clinical and morphological changes [3].

## Figures and Tables

**Table 1:** Clinical/sonographic ‘look’ on the Uhthoff cycle.

	Resting Phase	Resorptive Phase	Post-Calcific Phase
**Clinical** **Findings**	Mechanical pain movement-induced (e.g., subacromial/subcoracoid impingement)	Inflammatory pain poorly dependent on movement (e.g., acute microcristalline bursitis)	Mixed pain coupled with glenohumeral stiffness (e.g., adhesive capsulitis)
**Sonographic** **Findings**	Hard, arc-shaped calcific plaque with complete acoustic shadowing	Soft, irregular calcification with incomplete or absence acoustic shadowing	Regular echotexture of the rotator cuff tendons (no tendon sequelae)
**US-guided** **Procedures**	Perforation/needling of the hard plaque + Rehabilitation	Barbotage of the soft deposition and/or bursal injection + Rehabilitation	Glenohumeral injection ^§^ + Rehabilitation (+/− suprascapular nerve block)

^§^ anterior approach through the rotator interval or posterior approach through the fibrocartilage/labrum.

## Data Availability

Not applicable.

## Supplementary Materials

The following supporting information can be downloaded at: https://www.mdpi.com/article/10.3390/diagnostics12123097/s1, Supplementary Figure S1: A small fragment of the calcification (*void arrowhead*) is migrated inside the antero-medial recess of the subacromial-subdeltoid bursa, over the subscapularis (*SSC*) muscle-tendon unit. Note that the primary calcification from which the fragment is detached is not visible in this US image. Del, deltoid muscle; GT, greater tuberosity; LT, lesser tuberosity; Cor, coracoid; asterisk, long head of the biceps (hypoechoic due to anisotropy).
