# Supplementary figures and images for: Clinical/Sonographic Assessment and Management of Calcific Tendinopathy of the Shoulder: A Narrative Review
Source: Diagnostics (Basel). 2022 Dec 8;12(12):3097. doi: 10.3390/diagnostics12123097 (PMC9776939; doi:10.3390/diagnostics12123097)

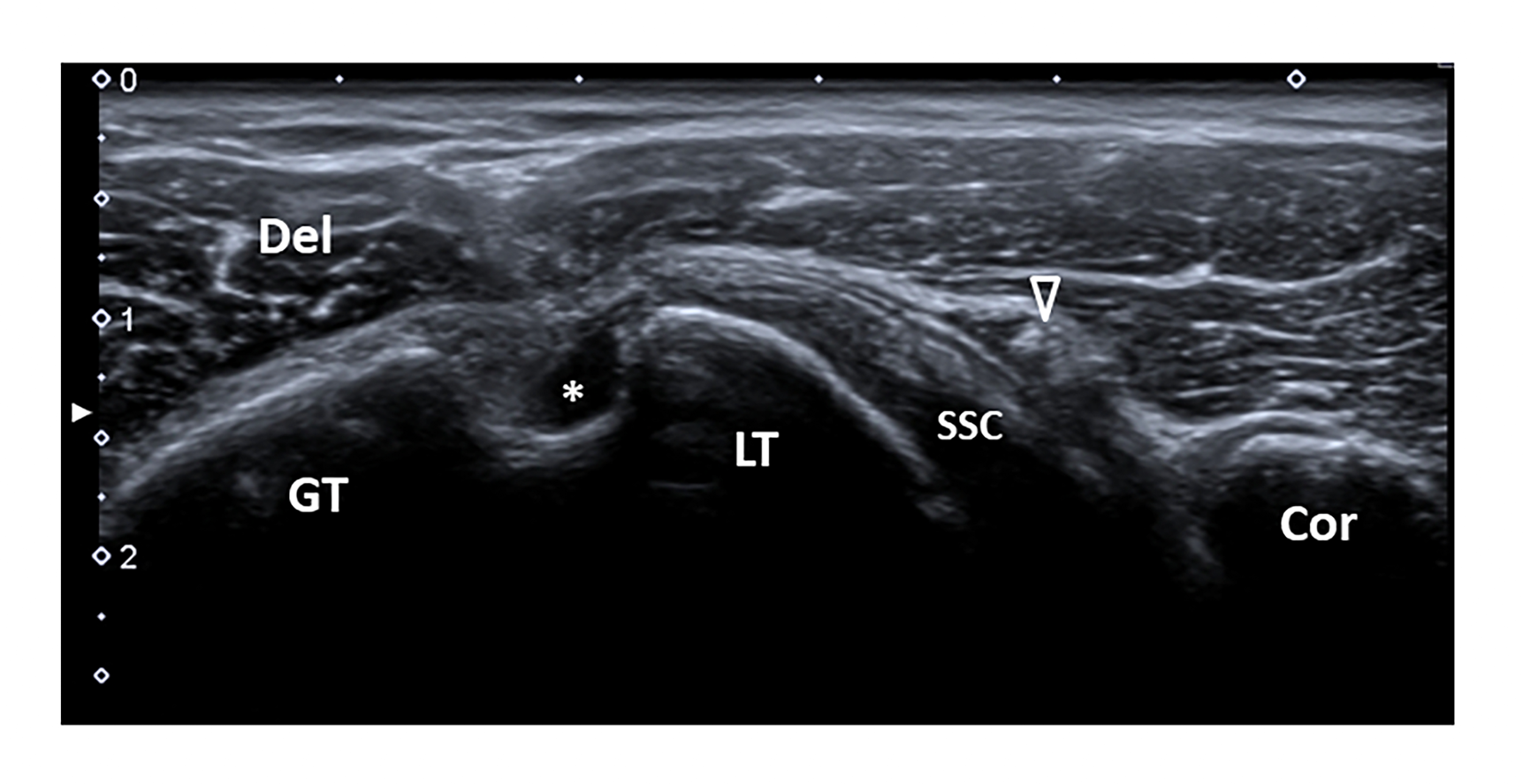

Supplement: Supplementary file 1 [file diagnostics-12-03097-s001.zip › diagnostics-2057461 Supplementary/Supplementary Figure S1.tif]
